# Evaluating the Guiding Role of Elevated Pretreatment Serum Carcinoembryonic Antigen Levels for Adjuvant Chemotherapy in Stage IIA Colon Cancer: A Large Population-Based and Propensity Score-Matched Study

**DOI:** 10.3389/fonc.2019.00037

**Published:** 2019-02-13

**Authors:** Qi Liu, Yongqiang Huang, Dakui Luo, Sheng Zhang, Sanjun Cai, Qingguo Li, Yanlei Ma, Xinxiang Li

**Affiliations:** ^1^Department of Colorectal Surgery, Fudan University Shanghai Cancer Center, Shanghai, China; ^2^Department of Oncology, Shanghai Medical College, Fudan University, Shanghai, China; ^3^Department of Urology, Fudan University Shanghai Cancer Center, Shanghai, China

**Keywords:** carcinoembryonic antigen, adjuvant chemotherapy, stage IIA colon cancer, PSM, SEER

## Abstract

**Objective:** This study was to investigate guiding role of elevated pretreatment serum carcinoembryonic antigen (CEA) levels for ACT receipt in stage IIA colon cancer.

**Methods:** Eligible patients diagnosed with stage IIA colon cancer (*N* = 21848) were identified from the Surveillance, Epidemiology, and End Results (SEER) database between January 2004 and December 2010. Pearson's chi-squared tests, Cox proportional hazards regression models, and Kaplan-Meier methods were performed. Propensity score matching (PSM) was used to decrease the risk of biased estimates of treatment effect.

**Results:** Multivariate Cox analysis indicated that, in CEA-elevated group, receiving or not receiving ACT did not presented statistically CSS difference [hazard ratio (HR) = 0.940, 95% confidence interval (CI) = 0.804–1.097, *P* = 0.431]; in CEA-normal group, receiving or not receiving ACT also did not presented statistically CSS difference (HR = 0.911, 95% CI = 0.779–1.064, *P* = 0.239). After PSM, Kaplan-Meier analyses showed that there was no statistical CSS difference between receiving or not receiving ACT (*P* = 0.64).

**Conclusion:** ACT did not show substantial survival benefit in stage IIA colon cancer with elevated pretreatment serum CEA levels. Stage IIA disease with elevated pretreatment serum CEA should not be treated with ACT.

## Introduction

Colon cancer is one of the most commonly diagnosed cancers in men and women (1). It was reported that stage II disease accounted for ~36% of new colon cancer diagnoses (2). The use of adjuvant chemotherapy (ACT) in stage II colon cancer was still controversial though it was widely accepted as standard treatment for patients with stage III colon cancer (3–5).

Although lack of enough direct evidence regarding the efficiency of ACT in stage II colon cancer, the American Society of Clinical Oncology (ASCO) clinical guidelines still had recommendations of ACT the so-called high-risk stage II disease (6). Also, the European Society for Medical Oncology (ESMO) had the similar recommendation for high-risk stage II colon cancer (7). In spite of a little different from each other regarding the definition of “high-risk factors,” both of ASCO and ESMO did not rank elevated serum carcinoembryonic antigen (CEA) levels as one of the so-called high-risk factors. And the ASCO Tumor Marker Panel had suggested that there was not sufficient data to support the use of preoperative CEA levels to guide the receipt of ACT stage II tumor (8). Later however, some reports supported the use of serum CEA levels to guide ACT (8–12) and regarded elevated preoperative CEA levels as one of the high-risk factors in stage II disease (13–16).

In fact, the efficacy of adjuvant ACT among high-risk stage II colon cancer had long been controversial (17–19). In spite of this, recently, the efficacy of ACT in T4 (stage IIB and IIC) disease was confirmed (18, 20, 21). In the present study, therefore, we have evaluated the guiding role of elevated pretreatment serum CEA levels for ACT receipt in stage IIA (T3N0M0) colon cancer using the large Surveillance, Epidemiology, and End Results (SEER) database.

## Patients and Methods

### Patient Selection From SEER Database

Data from the SEER Program of the United States National Cancer Institute were used. As an authoritative source, SEER collects patient demographic information, cancer diagnostic information, and outcomes from 18 cancer registries in the United States, thus including ~28% of the US population. The SEER database does not contain any identifiers and is publicly available for the studies of cancer-based epidemiology. In the present study, the National Cancer Institute's SEER-Stat software (version 8.3.5) was used to get access to SEER database.

**Figure 1:**
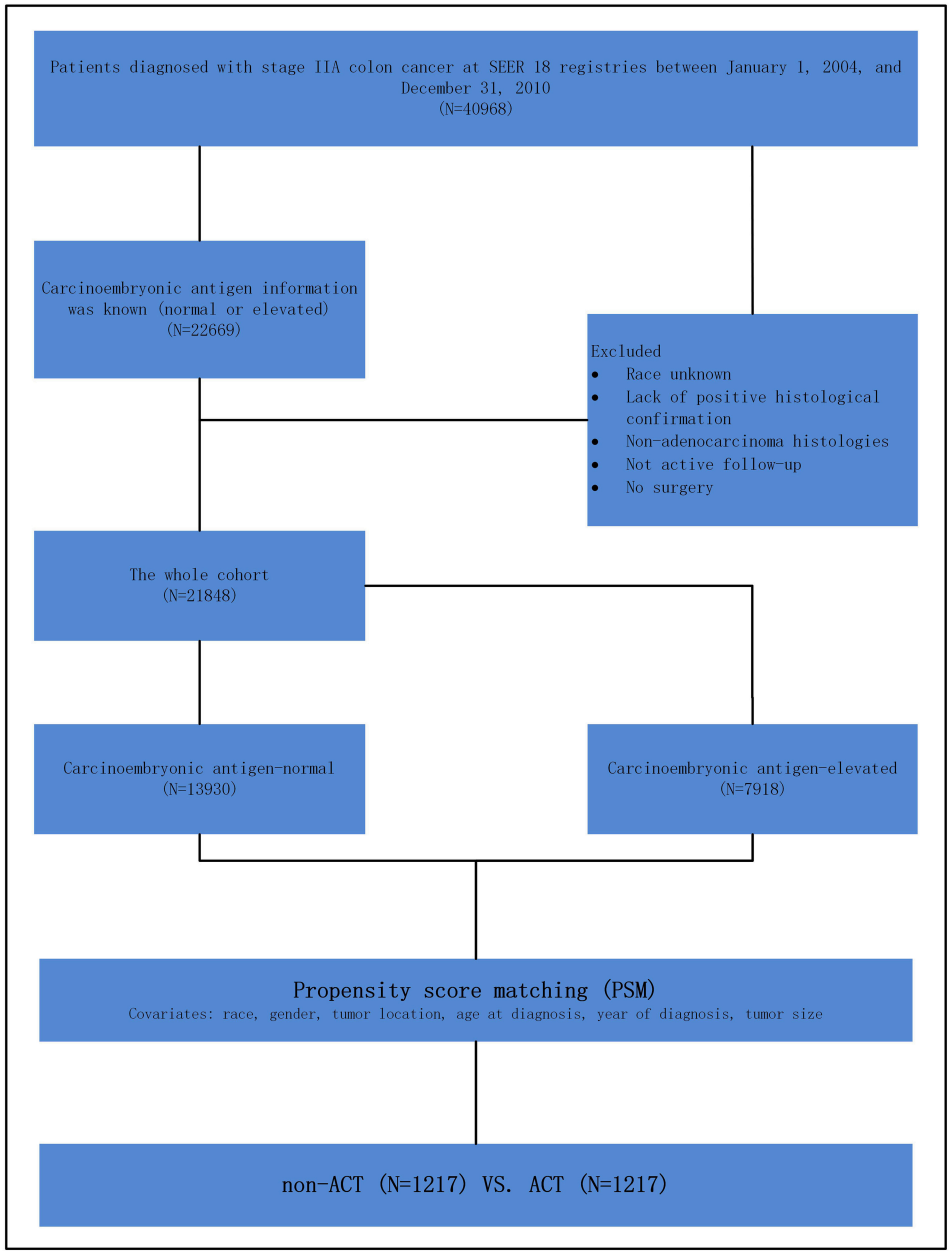
Schematic representation of patient population selected from Surveillance, Epidemiology, and End Results (SEER) database.

Shown as Figure 1, at first, 40,968 patients, diagnosed with stage IIA (T3N0M0) colon cancer between January 1, 2004 and December 31, 2010, were identified. We identified patients diagnosed within these years as pretreatment serum CEA information was recorded starting from 2004 and we wanted to allow for 5 years of follow-up (SEER follow-up ended in 2015).

Those with known CEA information were included in our study. The exclusion criteria were followed: race unknown, lack of positive histological confirmation, non-adenocarcinoma histology, not active follow-up, no surgery. In this study, we stratified the “patient had chemotherapy” as ACT group and “no evidence of chemotherapy was found in the medical records examined” as a non-ACT group in the variable “chemotherapy recode” in SEER cohort.

### Statistical Analyses

In the present study, we compared different clinicopathologic factors between the ACT and non-ACT groups using Pearson's chi-squared test for different variables. The outcome of interest in our study was cause-specific survival (CSS). The cause of death was categorized as colon cancer specific or non-colon cancer related. The CSS was calculated from the date of diagnosis to the date of colon cancer death. Patients who died of other causes were censored at the date of death. To determine whether there was a significant interaction between the level of serum CEA and ACT in predicting CSS, we defined a variable combined with serum CEA level and ACT. The Kaplan-Meier method with a log-rank test was used to analyze CSS. Then, several multivariate Cox proportional hazard models were constructed to identify independent prognostic factors along with hazard ratio (HR) for CSS. Variables that showed prognostic significance (log-rank, *P* < 0.20) in univariate analysis were included in the final multivariable analysis. Pair-wise comparisons were performed between different combinations of tumor grade and ACT to determine the presence of significant CSS differences.

However, as an observational one, there could be significant bias introduced by inherent differences between patients based on the receipt of ACT in the present study. Then, to decrease the risk of biased estimates of treatment effect, we defined the logit of predicted probability of treatment as a propensity score using the following patient and tumor characteristics: race, gender, tumor location, age at diagnosis, year of diagnosis and tumor size. Patients receiving ACT were matched on a one-to-one basis with patients not receiving ACT. Patients with and without receiving ACT were matched within their respective risk groups, matching was performed based on nearest-neighbor matching. Propensity scores reflect the probability that patients received or not received ACT based on their baseline characteristics. The details of propensity score matching (PSM) process are shown as Figure 1.

Statistical analysis was mainly performed with SPSS version 22 (IBM Corporation, Armonk, NY, USA); and two-sided *P* < 0.05 was considered statistically significant.

## Results

### Baseline Characteristics

Twenty one thousand eight hundred and forty-eight patients diagnosed with stage IIA colon cancer were identified from SEER database with a median follow-up time of 75 months. And 2,666 (12.2%) patients died of colon cancer at the end of the follow-up time. Of all, 13,930 patients (63.8%) were stratified into the CEA-normal group, and 7,918 patients (36.2%) were stratified into the CEA-elevated group. The patients' baseline demographic characteristics are shown in Table 1. The results of Pearson's chi-squared test indicated that the level of serum carcinoembryonic antigen was not corrected with the receipt of ACT (*P* = 0.823, Table 1).

**Table 1:** Comparison of baseline characteristics of the whole cohort by receipt of ACT.

**Variable**	**No. of Patients (%)**		***P***
	**Non-ACT (*N* = 18,368)**	**ACT (*N* = 3,480)**	
Race			0.151
White	15,055 (82.0)	2,805 (80.6)	
Black	1,960 (10.7)	394 (11.3)	
Other	1,353 (7.4)	281 (8.1)	
Gender			0.025
Male	8,925 (48.6)	1,763 (50.7)	
Female	9,443 (51.4)	1,717 (49.3)	
Tumor location			< 0.001
Cecum	4,361 (23.7)	673 (19.3)	
Ascending colon	4,427 (24.1)	656 (18.9)	
Hepatic flexure	1,331 (7.2)	213 (6.1)	
Transverse colon	2,236 (12.2)	406 (11.7)	
Splenic flexure	785 (4.3)	178 (5.1)	
Descending colon	1,138 (6.2)	272 (7.8)	
Sigmoid colon	4,090 (22.3)	1,082 (31.1)	
CEA			0.823
Elevated	11,717 (63.8)	2,213 (63.6)	
Normal	6,651 (36.2)	1,267 (36.4)	
Age at diagnosis (years)			< 0.001
< 60	2,929 (15.9)	1,603 (46.1)	
60–69	3,692 (20.1)	1,030 (29.6)	
70–79	5,605 (30.5)	691 (19.9)	
80+	6,142 (33.4)	156 (4.5)	
Year of diagnosis			< 0.001
2004–2007	10,357 (56.4)	2,176 (62.5)	
2008–2010	8,011 (43.6)	1,304 (37.5)	
Tumor size			< 0.001
≤ 5 cm	10,968 (59.2)	1,866 (53.6)	
>5 cm	6,962 (37.9)	1,498 (43.0)	
Unknown	538 (2.9)	116 (3.3)	

*ACT, adjuvant chemotherapy*.

### Associations of the Level of Serum CEA and ACT in Predicting CSS

**Figure 2:**
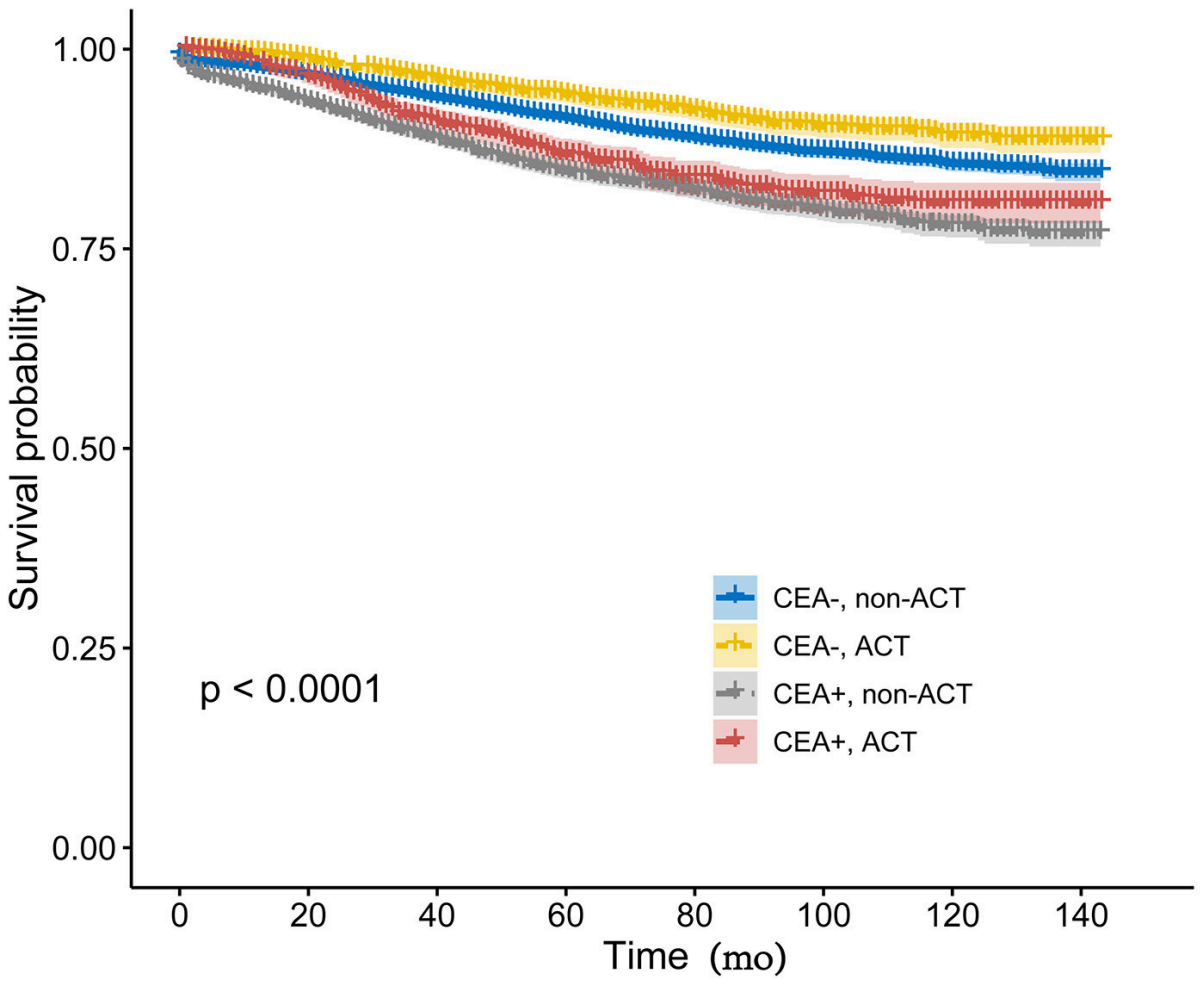
Kaplan-Meier CSS curves stratified by the combination of pretreatment serum CEA levels and receipt of ACT.

Kaplan-Meier analysis showed that the receipt of ACT had better 6-year CSS (median follow-up time was 75 months) rate compared with not receiving ACT with serum CEA elevated (84.8 vs. 83.3%, *P* = 0.026, Figure 2). Similarly, in the context of normal serum CEA, the receipt of ACT had better 6-year CSS rate compared with not receiving ACT (93.0 vs. 89.6%, *P* < 0.001, Figure 2). After adjusting for other prognostic factors, such as race, gender, tumor location, age at diagnosis, and year of diagnosis, however, the results of multivariate Cox analysis indicated that, in CEA-elevated group, receiving or not receiving ACT did not presented statistically CSS difference (HR = 0.940, 95% CI = 0.804–1.097, *P* = 0.431). Similarly, in the pair-wise comparison, receiving or not receiving ACT also did not presented statistically CSS difference with normal serum CEA (HR = 0.911, 95% CI = 0.779–1.064, *P* = 0.239; Table 2).

**Table 2:** Multivariate Cox regression analyses of CSS of the cohort.

**Variable**	**Overall**		**Pairwise**	
	**HR (95%CI)**	***P***	**HR (95%CI)**	***P***
Race		<0.001	…	…
White	Reference			
Black	1.409 (1.257–1.579)	<0.001		
Other	0.939 (0.809–1.090)	0.411		
Gender		<0.001	…	…
Male	Reference			
Female	0.821 (0.760–0.887)			
Tumor location		<0.001	…	…
Cecum	Reference			
Ascending colon	0.903 (0.804–1.105)	0.087		
Hepatic flexure	0.929 (0.781–1.104)	0.401		
Transverse colon	0.947 (0.822–1.091)	0.452		
Splenic flexure	1.323 (1.102–1.589)	0.003		
Descending colon	1.156 (0.978–1.366)	0.089		
Sigmoid colon	1.333 (1.195–1.486)	<0.001		
Age at diagnosis (years)		<0.001	…	…
< 60	Reference			
60–69	1.334 (1.171–1.518)	<0.001		
70–79	1.728 (1.528–1.956)	<0.001		
80+	2.623 (2.315–2.974)	<0.001		
Year of diagnosis		0.059	…	…
2004–2007	Reference			
2008–2010	0.925 (0.853–1.003)			
CEA and the receipt of ACT		<0.001	…	…
CEA–, non-ACT	0.572 (0.491–0.667)	<0.001	Reference	
CEA–, ACT	0.521 (0.428–0.634)	<0.001	0.911 (0.779-1.064)	0.239
CEA+, non–ACT	0.940 (0.804–1.097)	0.431	1.642 (1.511-1.785)	<0.001
CEA+, ACT	Reference		1.748 (1.500-2.037)	<0.001

*CEA–, CEA-normal; CEA+, CEA–elevated*.

### CSS of ACT With Elevated Serum CEA After PSM

**Figure 3:**
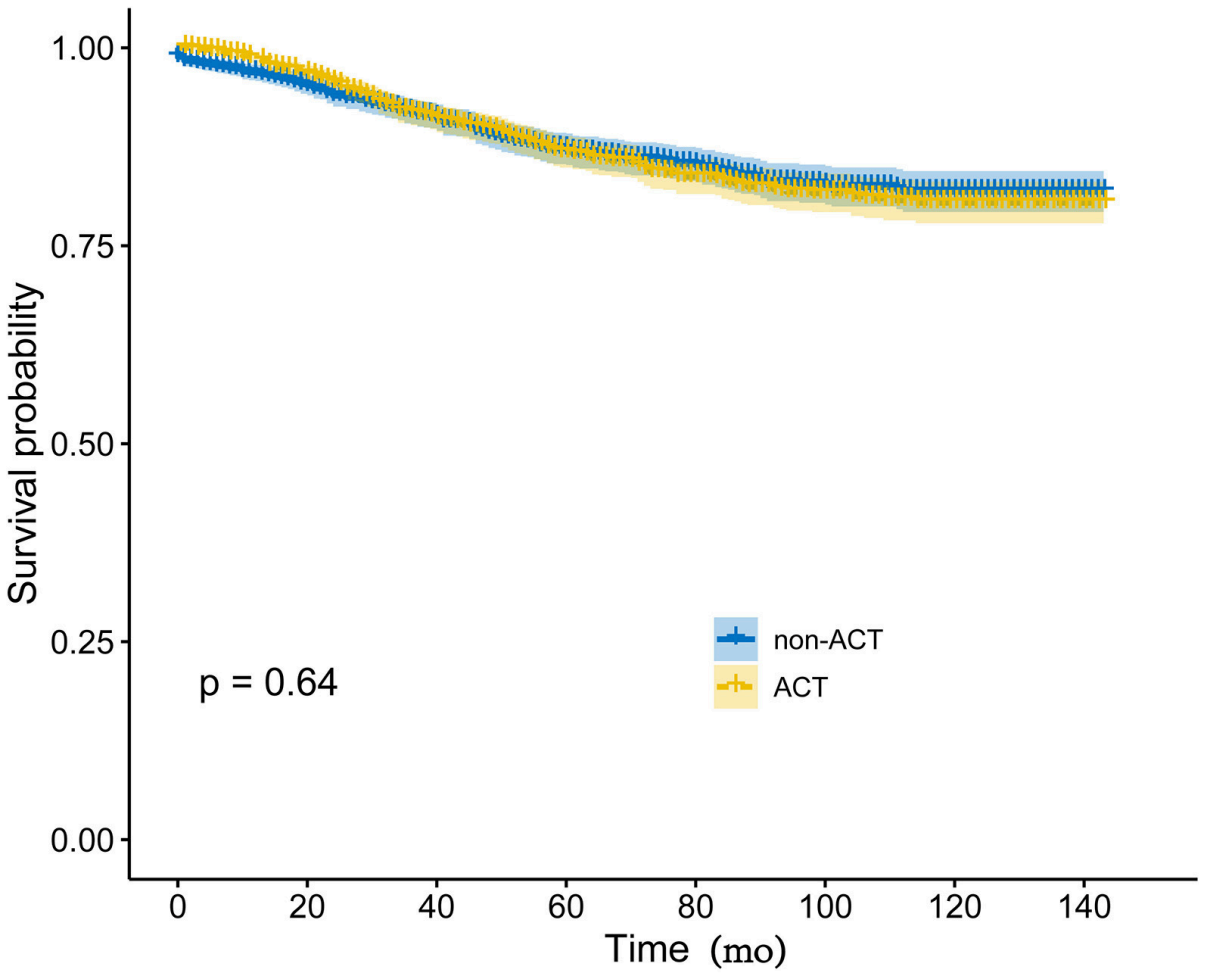
Kaplan-Meier CSS curves by the receipt of ACT in elevated pretreatment serum CEA group after PSM.

PSM produced 1,217 patients in the non-ACT group and 1,217 patients in the ACT group, all the tumor and patient characteristics showed no statistically differences between the two groups (Table 3). The Kaplan-Meier method with a log-rank test was used to compare the CSS difference of receiving and not receiving ACT after PSM, and the result indicated there was no statistical CSS difference between receiving or not receiving ACT (*P* = 0.64, Figure 3).

**Table 3:** Comparison of baseline characteristics of CEA-elevated stage IIA colon cancer by receipt of ACT after PSM.

**Variable**	**No. of Patients (%)**		***P***
	**Non-ACT (*N* = 1217)**	**ACT (*N* = 1217)**	
Race			0.674
White	943 (77.5)	958 (78.7)	
Black	175 (14.4)	160 (13.1)	
Other	99 (8.1)	99 (8.1)	
Gender			0.935
Male	607 (49.9)	605 (49.7)	
Female	610 (50.1)	612 (50.3)	
Tumor location			1.000
Cecum	242 (19.9)	240 (19.7)	
Ascending colon	228 (18.7)	229 (18.8)	
Hepatic flexure	143 (11.8)	67 (5.5)	
Transverse colon	68 (5.6)	142 (11.7)	
Splenic flexure	71 (5.8)	70 (5.8)	
Descending colon	90 (7.4)	93 (7.6)	
Sigmoid colon	375 (30.8)	376 (30.9)	
Age at diagnosis (years)			1.000
< 60	487 (40.0)	487 (40.0)	
60–69	387 (31.8)	387 (31.8)	
70–79	268 (22.0)	268 (22.0)	
80+	75 (6.2)	75 (6.2)	
Year of diagnosis			0.933
2004–2007	759 (62.4)	757 (62.2)	
2008–2010	458 (37.6)	460 (37.8)	
Tumor size			0.711
≤ 5 cm	592 (48.6)	596 (49.0)	
>5 cm	595 (48.9)	597 (49.1)	
Unknown	30 (2.5)	24 (2.0)	

*ACT, adjuvant chemotherapy*.

## Discussion

Stage II colon cancer had a relatively good prognosis, however, it was reported that ~15–30% of patients with stage II disease would eventually develop distant metastases or locoregional recurrent disease, resulting in poor outcomes even after resection of the primary tumor (2, 22). Furthermore, to avoid the potential of excessive treatment, identification of candidates in stage II colon cancer for additional therapy was imperative.

Although both ESMO and ASCO had the recommendation for high-risk stage II colon cancer to receive ACT, the efficacy of ACT in high-risk disease had long been controversial and was suspected by many studies (15–19, 21, 23). In spite of this, recently, the definite survival benefit of ACT had been reported in T4 disease (stage IIB and IIC) among stage II colon cancer patients (18, 20, 21). In 2014, Aalok and his colleges (20) reported that the recurrence-free survival (RFS), disease-specific survival (DSS), and overall survival (OS) benefit of adjuvant CT was mainly observed in the T4 disease. Two later studies from the USA (20) and Netherlands (21) revealed the same findings, thus proving that the maximum survival benefit in T4 can be obtained with adjuvant therapy.

As a 201 kDa highly glycosylated antigen, serum CEA is the single most important tumor marker and elevated preoperative CEA correlate with poorer prognosis in rectal cancer (24–26). In 2,000, the Colorectal Working Group of the American Joint Committee on Cancer (AJCC) even proposed the inclusion of serum level of CEA (C-stage) into conventional TNM staging of rectal cancer (27). Furthermore, the ASCO (25) and the European Group on Tumor Markers (24) have both recommended the use of preoperative serum CEA as a prognostic tool in rectal cancer. However, we noted that both ESMO (including lymph nodes sampling <12; poorly differentiated tumor; vascular or lymphatic or perineural invasion; tumor presentation with obstruction or tumor perforation and pT4 stage) and ASCO (including patients with inadequately sampled nodes, T4 lesions, perforation, or poorly differentiated histology) did not regard elevated serum CEA levels as one of high-risk factors of stage II colon cancer (6, 7). Recently, however, many reports supported the use of serum CEA to guide ACT in stage II colon cancer. We then conducted this large propensity score-matched study to evaluate the value of elevated serum CEA levels in a subset (stage IIA colon cancer, non-T4 disease) of stage II colon cancer which, as far as we knew, had never been reported before.

In this present study, univariate analyses showed little survival benefit offered by ACT in stage IIA colon cancer both with and without elevated pretreatment serum CEA levels. After controlling for other known prognostic factors, however, the survival differences of receiving and not receiving ACT were not statistically significant in both CEA-elevated and CEA-normal groups. Furthermore, PSM was used to consolidated our finding, and analyses after PSM once again demonstrated ACT did not substantially improve survival in stage IIA colon cancer with elevated serum CEA levels and elevated serum CEA should not be used as one of high-risk factors to guide ACT, which was consistent with the ASCO and ESMO recommendations (6, 7) and added a strong evidence to the view of the ASCO Tumor Marker Panel that there was not sufficient data to support the use of preoperative CEA levels to guide the receipt of ACT stage II tumor (8). And we also noted that, in our study, the level of serum CEA was not corrected with the receipt of ACT, indicating that elevated serum CEA was not used for guiding ACE in clinical practice of stage II colon cancer in US, which was consistent with the ASCO and ESMO recommendations.

Considering the definite efficacy of ACT in T4 disease, we then assumed that the primary reason that the finding of our study was conflicted with previous researches (8, 9) which showed the survival benefit of ACT in stage II colon cancer with elevated serum CEA was because the studies did not exclusively focus on stage IIA colon cancer and their study cohorts were mixed with the subset of stage IIB (T4aN0M0) and IIC (T4bN0M0) colon cancers.

The main strength of our study was that to the best of our knowledge, it was the first to evaluate the value of serum CEA levels for guiding ACT in stage IIA colon cancer. By using a large population-based registry, it was possible to detect the absolute survival difference between CEA-elevated and CEA-normal patients. Furthermore, we also used PSM to demonstrated our finding that ACT did not show statistically survival difference in stage IIA colon cancer with elevated serum CEA levels, which provided information to guide ACT in stage II colon cancer.

There are some limitations in our study. First, this study did not include some prognostic factors of colon cancer. Nowadays, molecular biomarkers that could affect the prognosis of stage IIA colon cancer such as microsatellite instability (MSI) and BRAF V600E mutation that have been intensively studies, were not included into our analyses, which might result into bias to some extent (28). Second, it was not possible to differentiate the type of CT, preoperative CT or postoperative CT in SEER database. Yet, as the preoperative CT is not the standard treatment for stage IIA disease, we could cautiously describe the “patient had chemotherapy” in “chemotherapy recode” variable as “ACT” receipt. Finally, the present study was retrospective rather than prospective, thus our conclusions still need to be validated in other cohorts, especially in large RCTs.

In conclusion, ACT did not show substantial survival benefit in stage IIA colon cancer with elevated pretreatment serum CEA levels. Our study provided a strong evidence that serum CEA did not has a guiding role for ACT in stage IIA colon cancer and stage IIA disease with elevated pretreatment serum CEA should not be treated with ACT.

## Ethics Statement

The study was approved by the Ethical Committee and Institutional Review Board of the Fudan University Shanghai Cancer Center. The data did not include the use of human subjects or personal identifying information and no informed consent was required for this study.

## Author Contributions

XL and YM conceived this study. QiL and DL improved the study design and contributed to the interpretation of results. SZ collected the data. QinL performed data processing and statistical analysis. QiL and DL wrote the manuscript. YH revised the manuscript. XL, YM, and SC approved the final version.

### Conflict of Interest Statement

The authors declare that the research was conducted in the absence of any commercial or financial relationships that could be construed as a potential conflict of interest.
